# The Utility of Malaria Rapid Diagnostic Tests as a Tool in Enhanced Surveillance for Malaria Elimination in Vanuatu

**DOI:** 10.1371/journal.pone.0167136

**Published:** 2016-11-30

**Authors:** Wesley Donald, Cielo Pasay, Jean-Olivier Guintran, Harry Iata, Karen Anderson, Johnny Nausien, Karryn J Gresty, Norman C. Waters, Lasse S. Vestergaard, George Taleo, Qin Cheng

**Affiliations:** 1 Malaria & Vector Borne Diseases Control (MVBDC), Ministry of Health, Port Vila Vanuatu; 2 Drug Resistance and Diagnostics, Australian Army Malaria Institute, Brisbane, Australia; 3 Clinical Tropical Medicine, QIMR-Berghofer Medical Research Institute, Brisbane, Australia; 4 World Health Organization, Vanuatu Country Office, Port Vila, Vanuatu; 5 Walter Reed Army Institute of Research, Malaria Vaccine Branch, Military Malaria Research Program, Silver Spring, Maryland, United States of America; 6 World Health Organization, Western Pacific Reginal Office, Manila, Philippines; Université Pierre et Marie Curie, FRANCE

## Abstract

**Background:**

As part of efforts to eliminate malaria, Vanuatu has piloted the implementation of enhanced malaria surveillance and response strategies since 2011. This involves passive case detection (PCD) in health facilities, proactive case detection (Pro-ACD) and reactive case detection (Re-ACD) in communities using malaria rapid diagnostic tests (RDTs). While RDTs improve case management, their utility for detection of malaria infections in ACDs in this setting is unclear.

**Methods:**

The utility of malaria RDTs as diagnostic tools was evaluated in PCD, in five rounds of Pro-ACDs and five rounds of Re-ACDs conducted in Tafea and Torba Provinces between 2011 and 2014. The number of malaria infections detected by RDTs was compared to that detected by PCR from collected used-RDTs.

**Results:**

PCD in Tafea Province (2013) showed a RDT-positive rate of 0.21% (2/939) and a PCR-positive rate of 0.44% (2/453), indicating less than 1% of suspected malaria cases in Tafea Province were due to malaria. In Pro-ACDs conducted in Tafea and Torba Provinces, RDT-positive rates in 2013 and 2014 were 0.14% (3/2145) and 0% (0/2823), respectively, while the corresponding PCR-positive rates were 0.72% (9/1242) and 0.79% (9/1141). PCR identified villages in both provinces appearing to be transmission foci with a small number of low-density infections, mainly *P*. *falciparum* infections. In five rounds of Re-ACD, RDTs did not identify any additional infections while PCR detected only one among 173 subjects screened.

**Conclusions:**

PCD and Pro-ACDs demonstrate that both Tafea and Torba Provinces in Vanuatu has achieved very low malaria prevalence. In these low-transmission areas, conducting Pro-ACD and Re-ACDs using RDTs appears not cost-effective and may have limited impact on interrupting malaria transmission due to the small number of infections identified by RDTs and considerable operational resources invested. More sensitive, field deployable and affordable diagnostic tools will improve malaria surveillance in malaria elimination settings.

## Introduction

Vanuatu is a South Pacific country with approximately 80 islands, and a population of about 260,000. The country is endemic for both *P*. *falciparum* and *P*. *vivax*, with higher malaria prevalence in northern provinces. Over the past decade the malaria incidence rate in Vanuatu has declined due to intensified control measures and the country met the set target of >75% decrease in incidence between 2000 and 2015 [1]. Today, Vanuatu is among a group of 36 countries implementing a malaria elimination program [2], initially with particular attention to Tafea province, which is located towards the south of Vanuatu and is comprised of five islands: Tanna, Futuna, Erromango, Aniwa and Aneityum. Among these islands Futuna was not endemic for malaria and Aneityum is considered malaria free after a successful malaria elimination campaign conducted in the early 1990s [3]. Prior to implementation of the malaria elimination program in Tafea province in 2008, the number of confirmed malaria cases per 1000 was 20.9 [4]. A baseline survey conducted in the same year revealed an overall point prevalence of 2.7% with considerable heterogeneity between different islands: Tanna had a range from 0 to 12%, Erromango approximately 0.5% and Aniwa 0% [5]. In 2011, the surveillance system recorded only 0.7 confirmed malaria cases per 1000 people in Tafea province [4], a 96% reduction compared to 2008.

Another province targeted for malaria elimination is Torba Province, which is located in the northern end of Vanuatu, comprising of the Torres Islands and the Banks Islands. Historically, Torba Province has had higher malaria incidence rates than Tafea Province. In 2011, there were 46.9 confirmed malaria cases per 1000 people in Torba province [4].

Since 2011, Vanuatu has piloted the implementation of enhanced case based surveillance, including active case detection (ACD) as part of the transition from control to elimination. This is, in addition to passive case detection (PCD), a strategy involving proactive case detection (Pro-ACD) and reactive case detection (Re-ACD) [6].

PCD is the detection of malaria cases among patients seeking treatment in a health facility, usually for febrile disease, for which a malaria diagnosis is confirmed by a parasitological test. Quality-assured microscopy and RDTs are equivalent in terms of performance for the diagnosis of uncomplicated malaria [7]. The Vanuatu Malaria Elimination guidelines recommended the use of RDTs for PCD in places where microscopy is not available [6]. This is in agreement with the recent WHO guidelines on surveillance for elimination settings [8].

ACD can be proactive or reactive. Pro-ACD is the detection by health workers of malaria infections at community and household level among population groups that are considered to be at high risk. It has been reported that a large fraction of *P*. *falciparum* and *P*. *vivax* infections in a population are asymptomatic [9,10,11,12], but remain infectious to mosquitoes although at lower success rates [13] [14]. These asymptomatic cases are typically missed if only PCD is practiced. Another problem is that a substantial portion of infections are of a parasite density below the sensitivity threshold for detection by microscopy or RDTs, and their proportions are higher in low and very low transmission settings [15,16]. Such low-density infections could remain asymptomatic for weeks and months, yet contribute to a significant portion of the malaria transmission in low-endemic settings [14,17]. Pro-ACD offers an opportunity to detect some of these asymptomatic low density infections, especially if highly sensitive diagnostic methods are used.

Re-ACD is case investigation for each notified case of confirmed malaria in the field (index case), which includes obtaining the details of the confirmed case, reviewing local transmission potential and conducting ACD in the vicinity of the case, which involves the testing of household members and neighbours of the index case [18]. During the elimination phase, ACD becomes crucial for targeting the asymptomatic parasite reservoirs.

Although ACD is recommended by the WHO [18], several technical and operational questions around ACD remain unanswered. For example, is ACD a cost-effective way to reduce malaria transmission when malaria is already at very low level; what is the most effective and feasible way to target populations with Pro-ACD; which is the most efficient diagnostic test to use, and what is the best strategy for the frequency and timing of screenings? For Re-ACD, in addition to questions regarding what diagnostic tools to use, the optimal timing for investigation after the report of index cases, and radius around the home of the index cases is also undefined [19]. Many different surveillance strategies are applied experimentally in various countries in the Asia-Pacific region [20] and elsewhere [21].

Malaria RDTs were introduced in Vanuatu to improve case management and surveillance as part of the intensified malaria control and elimination program launched in 2009. As *P*. *falciparum* and *P*. *vivax* coexist in Vanuatu, combo RDTs (pfHRP2/PAN pLDH) detecting both species were selected and deployed over the whole country. The first brand of RDT introduced in 2009 was the ICT^TM^ Combo test, but this brand was replaced by CareStart^TM^ in 2012 on the basis of higher sensitivity of the later brand, particularly in detecting *P*. *vivax*, as documented by the WHO-FIND malaria RDT product testing programme [22].

While quality RDTs are recommended and have been used as the standard diagnostic tool for routine malaria case management and PCD, it is unclear whether RDTs are useful tools in surveillance at low transmission settings where a substantial proportion of low density and sub-microscopic infections were reported [15,16]. It is generally thought that more sensitive methods, such as PCR [11] and loop mediated isothermal amplification (LAMP) [23,24,25], are required for ACD to screen populations harbouring low density and sub-patent infections. However, very few field studies have shown the proportion of malaria infections RDTs can detect in routine surveillance.

Molecular tests can be used to estimate the total number of, and the prevalence of malaria infections in the community. These tests usually rely on extra blood collected, i.e. in addition to blood required for smears and RDTs, during the surveillance. In recent years, used-RDTs have been shown to be a useful source for DNA extraction for PCR [26,27]. Therefore, collection of used-RDTs provides a convenient way for sample collection, and thus a system of PCR based on routinely collected used-RDTs could be instrumental as malaria surveillance tools and provide important epidemiological data on malaria transmission. In addition, secondary testing of used-RDTs with PCR could help to evaluate the performance of field-deployed RDTs.

In this paper, we evaluate the utility of RDTs as surveillance tools in PCD and ACDs conducted in two provinces in Vanuatu. This was achieved by comparing the number and prevalence of malaria infection detected by RDTs to those detected by PCR from used-RDTs that were collected during the same community surveys. The findings will inform future malaria elimination strategies.

## Materials and Methods

### Field sites and surveys

The surveillance activities described in this paper were conducted in Tafea and Torba provinces, which are located towards the southern and the northern part of Vanuatu, respectively. For all surveys conducted, a written informed consent was obtained from each participant. Information on name, residence, age and sex of the tested participants was recorded prior to testing. Following registration, participants had a finger prick performed with a blood lancet and a few drops of blood were collected for the malaria tests. RDT positive cases were investigated, clinically assessed and given appropriate treatment as per national guidelines.

**Tafea province**: The national malaria control program in Vanuatu has targeted Tafea Province for malaria elimination since 2008. Enhanced malaria case surveillance and response operations were implemented since 2011 as part of the transition from control to elimination. RDTs (CareStart^TM^ Malaria HRP2/pLDH (Pf/PAN) Combo G0131, AccessBio) were used in PCD, two rounds of Pro-ACDs and five rounds of Re-ACDs conducted in Tafea Province. Post purchasing, lot testing of RDTs were performed by the WHO-FIND malaria RDT lot testing laboratories.

1. 2013 PCD. In 2013, all patients presenting with fever to a number of health facilities on Tanna Island, including one hospital, 11 dispensaries, four health centres and 28 aid posts, were tested for malaria with RDTs.
2. 2013 Pro-ACD. In Nov 2013, a house-hold survey was conducted in 10 villages on Tanna Island. These villages were selected because of recent malaria incidences.
3. 2014 Pro-ACD. A house-hold survey covering one village on Erromango Island and eight villages on Tanna Island was conducted between 6 May and 18 Aug 2014. Four of the eight villages surveyed on Tanna Island were included in the 2013 Pro-ACD.
4. 2013 and 2014 Re-ACDs. Re-ACDs were conducted in different locations on Tanna Island, Tafea province in Vanuatu. Every RDT-positive case is classified as an index case which triggers a case investigation in the village of residence during which recent travel history is collected. Cases are classified as local if individuals did not report any overnight stay outside the village of residence 14 days prior to the diagnosis. All households within a 500 m radius of each index case that was notified to the provincial malaria team was visited by a survey team within 5 days after case notification. GPS coordinates were used to map the tested household. All individuals were finger-pricked for RDT testing and preparation of 2 blood spots dried on filter papers (≥20μL each). Four rounds of Re-ACDs were conducted in Nov 2013, during which 7 to 52 individuals were screened with a RDT at each Re-ACD. The fifth Re-ACD was conducted in Oct 2014 in Lounanen where a total of 47 individuals from 11 house-holds were screened.

**Torba province**: The national malaria program conducted three rounds of Pro-ACD surveys to detect malaria cases and point prevalence in selected islands in Torba Province.

1. The 2011 Pro-ACD. In Dec 2011, Pro-ACDs were conducted on two islands in the Torres Island group: Toga Island and Loh Island. ICT Malaria Combo Cassette test ML02 (ICT Diagnostics) was used in this survey. A total of 485 individuals were included in the survey; 292 individuals were registered on Toga Island and 193 individuals on Loh Island.
2. The 2013 Pro-ACD. In Aug 2013 a follow-up Pro-ACD was conducted on Toga Island and Loh Island, and also on Hiw Island and Tegua Island of the Torres Island group, Torba Province. A total of 891 individuals were screened with CareStart^TM^ Malaria HRP2/pLDH (Pf/PAN) Combo G0131 (AccessBio).
3. The 2014 Pro-ACD. Another house-hold survey was conducted between 18 Nov and 8 Dec 2014 on the Banks Island group of Torba Province including islands of Vanua Lava, Gaua, Motalava and Mota. A total of 1705 among 1827 registered residents (93.32% coverage) were screened with CareStart^TM^ Malaria HRP2/pLDH (Pf/PAN) Combo G0131 (AccessBio).

### Collection of used-RDTs

Used-RDTs were packed and stored with desiccant at room temperature until shipment to AMI in Brisbane, Australia.

#### Selection of used-RDTs for PCR

Used-RDTs were sorted and numbered. Approximately 50% of used-RDTs from each Pro-ACD were selected for PCR. However, when the total number of RDTs collected from a survey exceeded 1500, approximately 30% of RDTs were selected for PCR. Depending on the proportion of RDTs to be processed, between 1/3 and 1/2 of used-RDTs were randomly selected from each household or each village. 100% of used-RDTs from Re-ACDs were used for PCR because the total number of RDTs collected from Re-ACDs is relatively small.

#### Extraction of DNA from used-RDTs

The selected RDTs were disassembled, nitrocellulose strips removed, cut to small pieces (2–5 mm each) and placed in Eppendorf tubes. Genomic DNA was extracted from these pieces using QIAamp DNA Mini Kits and a QIAcube robot (QIAGEN, Crawley, UK) following manufacturer’s instructions. DNA was eluted in 100 μL volume.

### Determination of Plasmodium spp by PCR

A multiplex PCR amplifying the parasite 18s RNA gene was performed to determine the presence of *Plasmodium spp*. as previously described [11]. 5–15 μL of DNA was used in each 25 μL PCR reaction.

### Ethical clearance

The survey protocols were approved by the Vanuatu Ministry of Health Ethics Committee and by the WHO Western Pacific Regional Office Ethical Review Committee (2014.6.VAN.1.MVP). The storage and processing of samples for PCR at AMI was approved by Australian Defence Human Research Ethics Committee (ADHREC 505–07). All participants of the active surveys provided a written consent as per approved survey protocols.

#### Data analyses

Graphpad Prism version 6.05 was used for data analyses and graph production. The overall malaria point prevalence for Tafea and Torba province were calculated as the mean and 95% confidence intervals based on the village and island prevalence data. 95% confidence intervals for sensitivity and specificity of RDTs were calculated using modified Wald method.

## Results

### Malaria detection in surveys conducted in Tafea Province 2013–2014

#### Malaria detection in PCD, 2013

Between July-Dec 2013, 939 symptomatic patients on Tanna Island were tested for malaria using CareStart RDTs. Only two positive RDTs were observed: one positive for PAN band indicating non-*P*. *falciparum* (non-Pf) infection and one positive for both Pf/PAN indicating either *Pf* infection or a mixed species infection. Two additional cases were detected by microscopy (one Pv and one Pf). PCR of 453/939 (48%) used-RDTs which included two positive RDTs detected *P*. *falciparum* DNA from three RDTs: one of the two positive RDTs that was positive for Pf and two of the 451 negative RDTs. Assuming the same PCR positive rate applies to the remaining negative RDTs, the total number of malaria infections in 939 patients would be estimated to be 5 (4+1). This indicates that malaria was the cause in only 0.53% (5/939) of fever patients on Tanna Island, Tafea Province.

#### Malaria detection in Pro-ACD 2013

During the 2013 Pro-ACD, 1250 people were screened with RDTs. Two individuals were positive by RDT: one Pf band positive and one PAN band positive (non-*Pf*, but negative on 2^nd^ RDT), giving a point prevalence of 0.16% (2/1250). PCR of 713 used RDTs (57% of used RDTs) identified a total of 6 *P*. *falciparum* infections across 5 of the 10 villages, giving a point prevalence of 0.84% (6/713), which is 5.25 fold higher than that of RDTs (Table 1). Both positive RDTs were negative by PCR suggesting RDT false positivity.

**Table 1:** Time, location, number of individuals tested and results of RDT and PCR from used-RDTs in Pro-ACDs conducted in Tafea Province.

Month Year/RDT	Island/Village surveyed	RDT results				PCR results				
		No. RDTs	No. +ve RDTs	P% (95% CI)	Spp.	No. RDTs PCRed	% RDTsPCRed	No. PCR +ve	P% (95% CI)	Spp (No.)
Nov 2013 CareStart	Tanna/Bethel	125	0	0	-	70	56.00	0	0.00	-
	Tanna/Imaen	125	0	0	-	70	56.00	1	1.43	Pf (1)
	Tanna/Imaio	137	0	0	-	76	55.47	2	2.63	Pf (2)
	Tanna/Imaki	82	0	0	-	46	56.10	0	0.00	-
	Tanna/Isaka	85	1^^#^	1.19	PAN	48	57.14	1	2.08	Pf (1)
	Tanna/Lelualu	90	0	0	-	51	56.67	0	0.00	-
	Tanna/Lounapkamei	239	0	0	-	127	53.14	1	0.79	Pf (1)
	Tanna/Lounapkituan	43	0	0	-	24	55.81	0	0.00	-
	Tanna/Port Resolution	259	1^	0.39	Pf	163	63.18	1	0.61	Pf (1)
	Tanna/Yapkapen	67	0	0		38	56.72	0	0.00	-
**Total**		**1252**	**2**^	**0.16 (-0.12–0.43)**	**PAN (1) Pf (1)**	**713**	**56.95**	**6**	**0.75 (0.05–1.45)**	**Pf (6)**
May-Jun 2014 CareStart	Erromango/Ipota	242	0	0	-	121	50.00	0	0.00	-
	Tanna/Ieruareng	47	0	0	-	47	100.00	0	0.00	-
	Tanna/Lenakel Cove	25	0	0	-	25	100.00	0	0.00	-
	Tanna/Lonomei	22	0	0	-	22	100.00	0	0.00	-
	Tanna/Yakasewar	13	0	0	-	13	100.00	0	0.00	-
	Tanna/Yapkapen	16	0	0	-	16	100.00	0	0.00	-
Aug 2014 CareStart	Tanna/Imaio	253	0	0	-	127	50.20	**5**	3.94	Pf (3),Pv (1), Pm (1)
	Tanna/Isaka	178	0	0	-	95	53.37	**2**	2.11	Pf (2)
	Tanna/Port Resolution	326	0	0	-	167	51.23	0	0.00	
**Total**		**1118**	**0**	**0**	**-**	**633**	**56.62**	**7**	**0.67 (-0.41–1.76)**	**Pf (5), Pv (1), Pm (1)**

Note

^ The RDT was negative by PCR.

^#^ The individual was negative when tested again on a 2^nd^ RDT.

#### Malaria detection in Pro-ACD 2014

In the 2014 Pro-ACD, no positive RDTs were observed after screening 1118 people living in one village on Erromango Island and eight villages on Tanna Island. PCR from the 633 (57%) used-RDTs identified plasmodial DNA in 7 individuals (Table 1). Five of these individuals lived in Imaio village, whereas two lived in Isaka village on Tanna Island. Both of these villages also had two and one individuals, respectively, positive by PCR in the 2013 Pro-ACD (Table 1). This indicates that these villages have an ongoing low level of malaria transmission. The overall point prevalence in nine villages surveyed in 2014 was 0.67% by PCR.

### Malaria prevalence in Torba Province 2011–2014

#### Malaria detection in Pro-ACD 2011

House-hold surveys were conducted on Loh and Toga Islands using ICT combo RDT. 14/485 (2.89%) residents screened gave a positive RDT result: eight positive for Pf, four positive for PAN and two positive for Pf/PAN. However, only one of the 14 positive RDTs had detectable plasmodial DNA by PCR indicating 13 false positive RDT results. PCR detected *P*. *falciparum* DNA from 11 negative RDTs in 274 (56%) of used-RDTs screened, giving a point prevalence of 4.38% (Table 2).

**Table 2:** Time, location, number of individuals tested and results of RDT and PCR from used-RDTs in Pro-ACDs conducted in Torba Province.

Month Year / RDT	Island/Village	RDT results				PCR results				
		No. of RDTs	No. of +ve RDTs	P% (95% CI)	Spp.	No. RDTs PCRed	% RDTs PCRed	No. PCR +ve	P% (95% CI)	Spp (No.)
Dec 2011 /ICT	Loh/Lunhariki	193	6	2.74	Pf (4)^ PAN (1)^Pf/PAN (1)^	133	68.9	4	3.01	Pf (4)
	Toga/Litaw	292	8	3.11	Pf (4) PAN (3)^Pf/PAN (1)^	141	48.29	8	5.67	Pf (8)*
	**Total**	**485**	**14**	**2.93 (0.57–5.28)**	**Pf (4+4^)PAN (4)^Pf/PAN (2)^**	**274**	**56.49**	**12**	**4.34 (-12.56–21.24)**	**Pf (12)**
Aug 2013 CareStart	Loh/	222	1	0.45	PAN (1)^	133	59.91	1	0.75	Pf (1)
	Toga/	350	0	0	-	212	60.40	1	0.47	Pf (1)
	**Sub-total**	**573**	**0**	**0.22 (-2.63–3.08)**	**-**	**345**	**60.21**	**2**	**0.61 (-1.17–2.39)**	**Pf (2)**
	Hiw/	242	0	0	-	144	60.25	1	0.69	Pf (1)
	Tegua/	79	0	0	-	40	50.63	0	0	-
	**Total**	**893**	**1**	**0.11 (-0.25–0.47)**	**PAN (1)^**	**529**	**59.24**	**3**	**0.48 (-0.06–1.02)**	**Pf (3)**
Nov–Dec 2014 CareStart	Vanua Lava/Mosina	251	0	0	-	74	29.48	0	0.00	-
	Vanua Lava/Vuries	604	0	0	-	180	29.80	2	1.11	Pv (2)
	Gaua/Namesarai	285	0	0	-	85	29.82	0	0.00	-
	Mota Lava/Neremigman	389	0	0	-	116	29.82	0	0.00	-
	Mot/Sarawia	176	0	0	-	53	30.11	0	0.00	-
**Total**		**1705**	0	**0**	**-**	**508**	**29.79**	**2**	**0.22 (-0.39–1.11)**	**Pv (2)**

Note

*One of the 8 positives by PCR was also RDT positive for Pf

^ negative by PCR.

#### Malaria detection in Pro-ACD 2013

In the 2013 survey, the newly introduced CareStart RDTs detected a single PAN positive case among 893 (0.11%) individuals screened. This RDT was negative by PCR. PCR detected 3 *P*. *falciparum* infections from 529 negative RDTs (59% RDTs screened), giving a point prevalence of 0.57% (Table 2). PCR detected 2 *P*. *falciparum* infections from 345 individuals of Loh and Toga islands in 2013 giving a point prevalence of 0.61%, an 86% reduction compared to 2011.

#### Malaria detection in Pro-ACD 2014

The 2014 Pro-ACD identified zero positive RDTs after screening 1705 residents across five villages on Vanua Lava, Gaua, Mota Lava and Mota islands. PCR of 508 used-RDTs identified 2 *P*. *vivax* infections, giving a point prevalence of 0.39% (Table 2).

**Fig 1:**
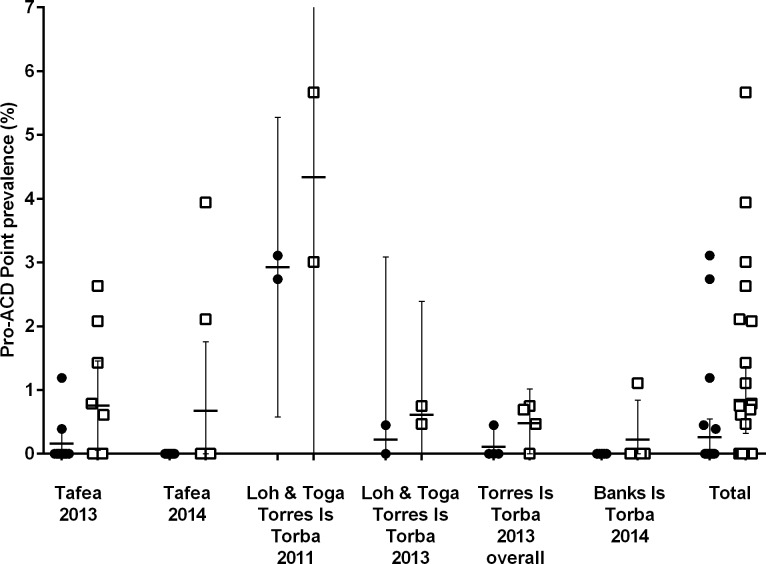
Comparison of RDT and PCR determined point prevalence (mean and 95% CI) of plasmodium infections in Pro-ACD. Filled circles represent RDT results while open squares represent PCR results.

In total, 5453 subjects from Tafea and Torba Provinces were screened with a RDT during five Pro-ACDs and RDTs identified 17 plasmodium infections of which 4 were confirmed by PCR. 2657 of the 5453 RDTs (48.73%) were tested by PCR, of which 30 RDTs were positive by PCR. The malaria prevalence in Tafea and Torba Provinces determined by RDTs and PCR from used-RDTs of the five Pro-ACDs is illustrated in Fig 1.

### Sensitivity and specificity of RDTs in Pro-ACDs

In the context of the Pro-ACDs, the sensitivity and specificity of the CareStart RDT compared to PCR was 0% (95% CI: 0–19.79%) and 99.88% (95% CI: 99.64–99.98%), respectively. The overall false positive rate is 0.12% (95% CI: 0.02–0.36%). The ICT RDT had a sensitivity and specificity of 8.33% (95% CI:<0.01–37.53%) and 95.04% (95% CI:91.62–97.15%), with a false positive rate of 4.74% (95% CI: 2.72–8.02%). The results suggest that RDTs are not sensitive at detecting the small number of asymptomatic infections in active surveillance.

### Re-ACD in Tafea Province 2013–2014

In 2013, four Re-ACDs were conducted on Tanna Island, Tafea Province. In each investigation, screening people around index cases within 5 days of their identification with a RDT did not detect any additional infections. PCR of all 126 used-RDTs from the four villages identified *P*. *falciparum* DNA in one individual who lived in Eniou village (Table 3). One Re-ACD was carried out in Lounanem village in 2014. The two index cases (both classified as local infections) were PAN band positive on RDTs and both RDTs were PCR positive for *P*. *vivax*. RDT screening did not detect any additional infections in 47 individuals (11 households) screened, nor did PCR from 47 used-RDTs (Table 3). Blood was also collected on filter papers from the same 47 individuals, and PCR from these filter papers did also not detect any additional infections.

**Table 3:** Time, location, number of individuals tested and results of RDT and PCR from used-RDTs in Re-ACDs conducted on Tanna Island, Tafea Province.

Year	Village on Tanna Island	Index case (By RDT)	RDT results			PCR results			
			No. of RDTs	No. +ve RDTs	Spp(No.)	No. RDTs PCRed	% RDTsPCRed	No. PCR +ve	Spp (No.)
2013	Isini	PAN	15	0	-	15	100	0	
	Eniou	PAN	52	0	-	52	100	1	Pf (1)
	Kitou	PAN	7	0	-	7	100	0	
	Lousula	Pf	52	0	-	52	100	0	
2014	Lounanen	PAN	49	2^#^	PAN (2^#^)	49	100	2	Pv (2^#^)

Note

^#^ indicates index cases.

## Discussion

The large scale deployment of RDTs for malaria case management has greatly improved access to confirmatory diagnosis in malaria endemic countries, contributing to the success of malaria control programs in recent years [1]. However, as countries implement enhanced surveillance in supporting their campaigns towards malaria elimination, the suitability of RDTs as surveillance tools in low transmission and malaria elimination settings becomes a key question. The focus of this study was to evaluate the utility of RDTs in enhanced malaria surveillance in Vanuatu by comparing the number of infections in communities detected by RDTs versus infections detected by PCR from used-RDTs in one round of PCD and several rounds of Pro-ACDs and Re-ACDs in different geographical locations. The outcomes shed light on the usefulness of RDTs in surveillance, as well as the usefulness of used-RDTs as sources of DNA for downstream molecular analysis that could produce epidemiological information to guide for malaria elimination activities.

In Vanuatu, malaria RDTs have played an important role in fever case management since 2009. During routine PCD conducted in health facilities on Tanna Island, Tafea Province in 2013, 939 fever patients were tested with a quality assured RDT that met the WHO procurement criteria. RDTs identified two malaria infections in 939 patients. PCR from 453/937 negative used-RDTs identified only a further two plasmodium infections indicating that only 0.53% (5/939) of fever in patients in Tafea Province was due to malaria and greater than 99% of fever cases should be investigated for other causes. Those patients missed by RDTs were likely to have parasite densities below the detection threshold of RDTs at the time, and may be detected by RDTs during follow up visits. The PCD results demonstrated a very low level of malaria incidence rate on Tanna Island.

RDTs and PCR from used-RDTs were also applied for detecting malaria cases in 5 rounds of Pro-ACD conducted in Tafea and Torba Province between 2011 and 2014. These Pro-ACDs all confirmed the low malaria transmission status in both Provinces. The overall malaria point prevalence detected during Pro-ACDs by RDTs for Tafea and Torba provinces was 0.16% and 0.11%, respectively in 2013, and 0% for both provinces in 2014. The corresponding point prevalence determined by PCR from used-RDTs was 0.75% and 0.48%, respectively, in 2013, and 0.67% and 0.22% in 2014. The higher detection rates by PCR indicated the presence of low density plasmodium infections in these communities, which is consistent with findings in similar settings [15,16]. Importantly, the results demonstrate clearly the success of the national malaria control and elimination program and confirm that both provinces have achieved very low levels of transmission.

Compared to PCR results, RDTs gave some false positive results: ICT produced 10 false positives in 485 tests performed in 2011, while CareStart gave three false positives in 2145 tests in 2013, 0 in 2823 tests in 2014. False positive *P*. *falciparum* on RDTs could have resulted from circulating PfHRP2 antigen of recent infections. It should also be noted that false positive Pf and PAN results could also relate to the false positive rate of the RDT product. For instance, the CareStart RDTs used since 2013 have a low false positive rate of 0.4%, which means that for every 1000 RDTs tested, 4 false positive results may be expected. The false positive rates observed in this study for this RDT were lower than 0.4%. Therefore, it would be a good practice that RDT positive results in Pro-ACDs are verified using quality microscopy. Confirmation of infection may trigger focal mass screening and treatment (MSAT) using a more sensitive diagnostic tool such as PCR or LAMP followed by treatment with ACT, or trigger deployment of alternative measures such as mass drug administration.

PCR from used-RDTs has helped to retrospectively detect possible transmission foci in both provinces. In Tafea Province, Imaio and Isaka villages on Tanna Island have more *Plasmodium* infections detected than other villages during 2013 and 2014, which may indicate active local transmission occurring around these villages. In Torba Province, it is likely that active transmission occurs in Loh and Toga islands as well as in Vuries village on Vanua Lava Island. These foci should be targeted for intensified surveillance and case investigations to confirm the presence of locally transmitted cases. Favouring factors that may contribute to the sustained transmission such as entomological, geographical, population, occupation and intervention coverage, should be investigated.

One interesting observation of the Pro-ACDs is the evidence of persistence of low density *P*. *falciparum* infections in both provinces. In Tafea Province, PCR of 713 used-RDTs from the 2013 Pro-ACD produced 6 *P*. *falciparum* positive results, and PCR of 633 used-RDTs from the 2014 Pro-ACD produced 5 *P*. *falciparum*, 1 *P*. *vivax* and 1 *P*. *malariae*. Combined, eleven of the 13 PCR positive infections were *P*. *falciparum*. This trend is consistent with the 2008 baseline prevalence survey in Tafea Province, where 68% of microscopy negative/PCR positive infections were *P*. *falciparum* [5], although the prevalence of infections has markedly decreased compared to 2008. A similar trend is observed in Torba Province. In the Pro-ACDs conducted in 2011, 2013 and 2014 *P*. *falciparum* was identified by PCR in 12/12, 3/3 and 0/2 infections, respectively. These findings are consistent with the recently reported distributions of parasite densities in asymptomatic malaria in Southeast Asia where a higher proportion of very low density *P*. *falciparum* infections were identified by an ultra-sensitive PCR compared to *P*. *vivax* [28].

The value of RDT as a surveillance tool in Pro-ACD could be judged by its impact on reducing malaria burden, interrupting local malaria transmission and cost-effectiveness, all of which can be influenced by the transmission intensity, the dominant *Plasmodium* species and the proportion of asymptomatic and sub-patent infections. In relatively high transmission settings of Africa where *P*. *falciparum* dominates, RDTs have been reported to consistently provide higher parasite prevalence than microscopy [29], likely due to the persistence of PfHRP2 antigen in recently infected individuals. However, the impact on malaria burden and transmission reported after treating RDT positive individuals identified in Pro-ACD has been variable. On one hand, longitudinal Pro-ACD with repeated screening of households using RDTs and treating RDT positive individuals with ACT was shown to reduce malaria incidence in Southern Zambia where the baseline RDT positive rate was ~40% [30]. On the other hand, monthly active home visits with RDTs and treatment in other Zambian settings did not significantly reduce malaria prevalence (measured by RDT) or incidence in the community [31]. These different outcomes could be related to the proportion of low density infections in the population. In areas with low transmission intensity and a high proportion of sub-microscopic infections, the utility of RDTs as surveillance tools does not appear to have a significant impact on malaria transmission. This was confirmed in a recent report from Zanzibar, where two rounds of mass screening using RDTs and treatment of RDT positive individuals did not lead to reduced malaria incidence rate subsequently [32]. In that setting, the malaria prevalence measured by RDT was 0.2% in both rounds and by PCR was 2.5% and 3.8% in round 1 and round 2, respectively. Recently, the WHO has made a recommendation for not conducting mass screening and treatment or focal screening and treatment as interventions to interrupt malaria transmission [33].

In Tafea and Torba Provinces in Vanuatu, the overall RDT positive rates in 2013 and 2014 were 0.14% (3/2145) and 0% (0/2823), respectively. Despite using a good performing RDT, the RDTs detected less than 20% of plasmodium infections identified by PCR. Because very considerable efforts and resources were invested in organising and conducting these Pro-ACDs, and because very few infections were detected by RDTs in the target communities which accounted for only a small proportion of true infections as judged by PCR, it appears that RDTs used alone as surveillance tools for Pro-ACD in locations with very low point prevalence of malaria, such as Tafea and Torba Provinces, may add limited value to interrupting malaria transmission. Although positive RDTs in Pro-ACDs could potentially be used as a trigger for more intensified malaria surveillance and treatment activities, RDTs as surveillance tools to detect asymptomatic infections appear not cost-effective.

Our study showed that PCR from used-RDTs did increase the detection rate by 2–5 fold compared to RDT alone. It demonstrates that used-RDTs provide a valuable source of DNA for molecular epidemiological studies and that the results help to assess malaria transmission status and to identify transmission foci. In addition, used-RDTs can serve as a source for serological studies for estimating sero-prevalence and for monitoring changes in transmission intensity [34]. Therefore, in malaria elimination settings, used-RDTs should be collected on a routine basis where possible for these surveillance purposes. However, while PCR from used-RDTs could provide useful epidemiological information, the resources required to disassemble the RDTs, extract the DNA and perform PCR analysis will likely prohibit this method being used routinely in the field in most programmatic settings. Therefore, more simple, sensitive and easy-to-perform molecular based assays are needed for effective routine surveillance in malaria elimination settings.

This study has some limitations. Although the sensitivity of PCR from used-RDTs was reported to be identical to that from filter papers [27], it should be noted that, like PCR from filter papers, its sensitivity is limited by the volume of blood applied to the RDTs, which is 5 μL for ICT and CareStart RDTs. We were not able to evaluate the level of PCR sensitivity based on these two different sources of blood samples in this study because filter paper samples were only collected from a small subset of individuals and because the number of positive samples by PCR was only small. In addition, while on average 51.8% of RDTs collected from Pro-ACDs were assayed by PCR, the proportion of RDTs analysed for the 2014 Pro-ACD in Torba province was approximately 30%. Although the RDTs were randomly selected from each household and each village, PCR on this proportion of RDTs may not be optimal in detecting the malaria infections in this community.

RDTs have been used in several countries for Re-ACDs and demonstrated ability to detect additional infections located in households within 140m, 500m or 1 km radius of index cases [35,36,37]. Vanuatu also conducted five rounds of Re-ACDs on Tanna Island, Tafea Province, which however proved highly demanding in terms of required preparations, manpower and other resources. In all five Re-ACDs, no additional subjects were found to be positive by RDTs and only one subject in one of the Re-ACDs was found positive by PCR. The results showed that family members living in the same households, or in the vicinity of the index cases were either not infected at the time of investigation or were below the detection threshold of the PCR method used. Subsequent visits to these households may thus be required to correctly identify secondary infections. The lack of positive cases detected by PCR prohibits our further analysis on the optimal size of Re-ACD “screening radius”, and whether RDTs had a sufficient sensitivity to detect additional cases; however, similar to recent findings by van Eijk et al [21], our results question the value and cost effectiveness of Re-ACD in areas with very low levels of malaria transmission.

## Conclusions

The use of RDTs in Pro-ACDs conducted in Tafea and Torba Province, Vanuatu revealed that malaria point prevalence rates have been brought down to at a level below 1% in both provinces since 2013. This was confirmed by retrospective PCR from used-RDTs. RDT and PCR nevertheless identified several possible malaria transmission foci in both provinces. However, in these very low transmission settings, implementation of Pro-ACD and Re-ACD using RDT as a detection tool to find asymptomatic malaria infections appears not to be cost effective and may have very limited impact on the overall effort towards interrupting malaria transmission. A simple, sensitive and easy-to-perform molecular diagnostic tool is required for effective malaria surveillance in malaria elimination settings.
